# Intensive Treatment in Adult Burkitt Lymphoma with Lymphome Malin B (LMB) Regimen: Excellent Outcomes Despite Substantial Toxicity and Supportive Care Demands

**DOI:** 10.3390/cancers17172914

**Published:** 2025-09-05

**Authors:** Ivan Dlouhy, Diana Viegas, Inês Coelho, Alina Ionita, Susana Carvalho, José Cabeçadas, Maria Gomes da Silva

**Affiliations:** 1 Hematology Department, Instituto Português de Oncologia de Lisboa Francisco Gentil, 1099-023 Lisboa, Portugal; 2 Pathology Department, Instituto Português de Oncologia de Lisboa Francisco Gentil, 1099-023 Lisboa, Portugal

**Keywords:** adult Burkitt lymphoma, immunochemotherapy, CNS infiltration

## Abstract

**Background**: Burkitt lymphoma is a rare, aggressive B-cell neoplasm with frequent central nervous system (CNS) involvement, treated with intensive multidrug regimens associated with rituximab. The aim of this study was to assess the efficacy, safety, and feasibility of the LMB protocol in adults with BL in a real-world setting. **Methods**: We included 55 patients with BL diagnosis according to the 2008 WHO classification, treated with LMB protocol associated with rituximab. Low-risk patients (no bone marrow or CNS involvement) were treated in the group B arm, while high-risk patients were placed in group C, which was further stratified by age and CNS infiltration. **Results**: Thirty-four patients (62%) were treated in group B and 21 patients (38%) were treated in group C, with a median age of 34 years (16–77). Extranodal infiltration was present in 71% patients, including 11 (20%) with CNS involvement. After a median follow up time of 7 years, the complete remission rate was 85%, and progression-free and overall survival at 3 years were 79% and 84%, respectively. Patients with CNS infiltration had an inferior survival rate (55% at 3 years). Grade 3–4 toxicities were frequent, mainly hematologic, infectious, and mucosal. Treatment required substantial supportive care, including 1604 transfusions and 4696 days of hospitalization. Patients over 60 years had poorer outcomes and higher toxicity. **Conclusions**: The LMB protocol demonstrated high survival rates in adult BL, although at the cost of significant toxicity and considerable health care resource utilization. Outcomes remained suboptimal in patients with CNS involvement despite treatment intensification.

## Simple Summary

Burkitt lymphoma (BL) is a rare, aggressive mature B-cell neoplasm with frequent central nervous system involvement. Treatment strategies usually include high doses of methotrexate and/or cytarabine associated with intensive chemotherapy and rituximab. Commonly used protocols such as R-CODOX-M/IVAC, Hyper-CVAD, and BFM differ in intensity, duration, and drug composition. While these approaches yield high response rates, they are also associated with substantial toxicity, prolonged hospitalization, and considerable transfusion requirements. Outside clinical trials, series are frequently small and heterogeneous, with few studies using the LMB protocol. We aimed to thoroughly examine a large series of adult BL patients treated in an experienced center with the LMB protocol, an intensive regimen utilized for aggressive lymphomas. This study gives valuable information about efficacy, toxicity and health resource utilization with this protocol, fueling the discussion about the best strategy for BL patients.

## 1. Introduction

Burkitt lymphoma (BL) is a rare, aggressive mature B-cell neoplasm with a variable incidence and epidemiology according to the geographical region and age, predominantly affecting males in a 2–4:1 ratio [1]. The defining genetic event that underlies BL lymphomagenesis is the constitutive *MYC* expression due to a reciprocate translocation between *MYC* and immunoglobulin genes. Among the three epidemiological variants recognized by the WHO classifications, sporadic BL is the most common in Western countries, representing 1–2% of all lymphoma cases in adults in Europe and North America.

Central nervous system (CNS) involvement is reported in 10–30% of patients with BL and constitutes a well-established adverse prognostic factor [2,3,4]. Consequently, most frontline therapies include CNS-directed agents with adequate blood–brain barrier penetration. Commonly used protocols, including R-CODOX-M/IVAC, Hyper-CVAD, LMB, DA-EPOCH-R and BURKIMAB, have demonstrated high response rates and durable remissions in both pediatric and adult populations, albeit at the cost of substantial toxicity and frequent need for prolonged hospitalization [5,6,7,8,9].

Data on BL management outside clinical trials is limited, heterogeneous, and often difficult to compare across series. While efficacy has been well established for all intensive treatment regimens, with similar high rates of disease control, the burden of toxicity and supportive care requirements, including transfusions and length of hospital stay, remains less well characterized [10].

For over 15 years, our center has employed a modified French Lymphome Malin B (LMB) protocol, a risk-adapted regimen that has been progressively optimized over time, including the incorporation of rituximab in its most recent version [7,11,12]. Risk stratification is based on CNS and bone marrow involvement, as well as patient age. In the trial incorporating rituximab (median age 47 years), a 3-year event-free survival (EFS) and overall survival (OS) of 75% and 83% were observed, respectively.

The aim of this study was to comprehensively assess the efficacy, safety, and feasibility of the LMB protocol (in association with rituximab) in adults with Burkitt lymphoma treated in a single, experienced cancer center.

## 2. Patients and Methods

### 2.1. Study Cohort

We retrospectively identified 63 BL patients diagnosed from January 2007 to December 2024 according to the World Health Organization (WHO) classification [13]. All consecutive patients older than 15 years old and treated with LMB protocol were analyzed. Six patients were treated with other regimens during this period and were excluded from the study. Two limited-stage, low-risk patients received the group A LMB95 protocol and were also excluded. Epidemiological, clinical, treatment and outcome data were retrieved from clinical records.

Staging procedures included patient history and physical examination; performance status according to the Eastern Cooperative Oncology Group (ECOG) scale, presence of B symptoms (fever, night sweats, weight loss) and bulky disease (defined as a tumor diameter ≥ 10 cm); complete blood cell counts; serum biochemistry, including lactate dehydrogenase (LDH), b2-microglobulin (b2m) levels and kidney and liver function tests; computerized tomography and/or positron emission tomography (PET-CT) scans; and unilateral bone marrow biopsy or aspirate. Lumbar puncture was performed in all patients during the pre-phase, while CNS imaging was conducted only when clinically indicated. CNS involvement was defined as cerebral-spinal fluid (CSF) infiltration, parenchymal lesions in CT/MRI or high clinical suspicion.

Disease was staged according to the Ann Arbor classification. International prognostic index (IPI) and BL-IPI, that includes age ≥ 40 years, PS ≥ 2, serum LDH over 3 fold the upper normal limit and CNS involvement, were calculated [14,15].

Response was assessed according to Lugano criteria after first consolidation and upon completion of chemoimmunotherapy [16]. OS was defined as the time from diagnosis to death from any cause. Progression-free survival (PFS) was defined as the time from diagnosis to disease progression, relapse, or death from any cause, whichever occurred first. Patients without events were censored at the date of last follow-up. Toxicities were classified according to the National Cancer Institute Common Terminology Criteria for Adverse Events (version 5, 2017).

### 2.2. Histological Review and FISH

All cases were classified according to the WHO classification available at the time of diagnosis, based on the integration of morphological, immunophenotypic, and genetic features [1,13,17]. In addition, all cases were reviewed by an expert pathologist. Histological examination was performed on formalin-fixed, paraffin-embedded (FFPE) tissue sections stained with hematoxylin and eosin. Immunohistochemical studies were performed on 3–5 μm FFPE tissue sections. The panel of primary antibodies included CD20, CD79a, CD10, BCL6, BCL2, CD38, and Ki-67 (Ventana Medical Systems, Roche, Basel, Switzerland). Additional markers such as MUM1, CD5, and TdT were used in selected cases for differential diagnosis as needed.

Detection of *MYC* gene rearrangement was performed by fluorescence in situ hybridization (FISH) using dual-fusion and/or break-apart probes (LSI t(8;14) cMYC-IGH CEP8 Tri-Color, Dual Fusion Translocation and MYC Dual Color, Break Apart Rearrangement; Vysis, Abbott, Chicago, IL, USA) according to the manufacturer’s protocol. At least 100 interphase nuclei were evaluated per case. Other probes used included dual fusion BCL2 (Dako FISH DNA Probe, Split Signal, Agilent Technologies, Santa Clara, Ca, USA), BCL6 (Dako, FISH DNA Probe, Split Signal) and Dual Color Dual Fusion LSI t(14;18) BCL2-IGH Translocation (Vysis). Cut-off value for positivity was 10% of analyzed nuclei.

Only cases with evidence of *MYC-IG* rearrangement and adequate morphology were considered consistent with classical BL.

### 2.3. Treatment Protocol

**Figure 1:**
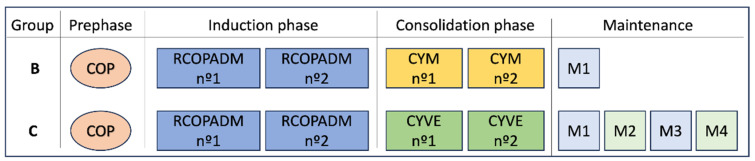
LMB protocol schedule.

Patients were treated according to the LMB protocol. Low-risk patients (without CNS or BM infiltration) were stratified into protocol group B, while high-risk patients received treatment with protocol group C (Figure 1), with variable doses of methotrexate (MTX) and cytarabine depending on age and CNS infiltration (Table S1). From 2007 to 2017, we used the LMB95 version of the protocol with the addition of rituximab in each cycle (five doses in group B and 8 doses in group C). Since 2017, patients have been treated using the LMB02 protocol version, which includes two dose-dense rituximab administrations during each induction R-COPADM cycle, for a total of four 375 mg/m^2^ doses.

Treatment for patients stratified into group B was identical across both protocol versions, except for the frequency of rituximab administration. On the other hand, in group C, besides the difference in rituximab administration, the third maintenance cycle (M3) in LMB95 did not include systemic high-dose MTX nor intrathecal chemotherapy. Also, doses of MTX and cytarabine were increased in the LMB02 protocol (Table S1).

Cycles were administered every 21 days with the exception of cytarabine-etoposide (CYVE) consolidations and maintenance, which were given every 28 days.

Hospitalization was mandatory during chemotherapy administration and optional during the neutropenic period for all cycles.

Prophylactic intrathecal chemotherapy consisted of 10 administrations of hydrocortisone plus methotrexate in group B and 7 to 11 administrations in group C with the addition of cytarabine (Table S2).

Cranial radiotherapy in patients with CNS infiltration was omitted in order to avoid additional neurological toxicity [18].

Interim staging was performed after the first consolidation cycle. Patients in group B who did not achieve complete response (CR) were escalated to group C, while group C patients without CR were rescued with platinum containing regimens. Patients who escalated to group C were analyzed as part of group B in all statistical analyses.

### 2.4. Statistical Analysis

Descriptive statistics were used to summarize patient characteristics. Continuous variables were reported as medians with minimum and maximum values and were compared between groups using Student’s t-test or Wilcoxon rank-sum test, as appropriate. Categorical variables were compared using the chi-squared test or Fisher’s exact test when expected frequencies were <5 cases.

Survival outcomes were assessed using the Kaplan–Meier method, with differences between groups compared using the log-rank test.

All analyses were conducted using R (version 4.4.2) within RStudio (Posit Software, Boston, MA, USA). A two-tailed *p*-value < 0.05 was considered statistically significant. Adjustment for multiple comparisons was performed using the Bonferroni method where applicable.

## 3. Results

### 3.1. Patient Characteristics

Patient characteristics at diagnosis are detailed in Table 1. Median age was 34 years, with 9 patients being older than 60 years. Most patients had a good ECOG performance status (PS) and a third of the patients had bulky disease at presentation. Extranodal involvement was frequent (71%), with the most affected localization being gastrointestinal. CNS infiltration was observed in 11 cases (20%) at diagnosis, and only 3 patients were HIV positive. No other causes of immunosuppression were identified.

No significant differences were observed between patients treated with LMB95 (*n* = 31) and LMB02 (*n* = 24) protocols in terms of initial clinical features, treatment group distribution, survival, days of hospitalization and toxicity.

### 3.2. Treatment

A total of 364 treatment cycles were administered. All but four patients received a cycle of cyclophosphamide, vincristine and prednisone (COP) as debulking therapy. Three patients received R-CHOP instead: two due to an initial misdiagnosis of large B cell non-Hodgkin lymphoma, and one diagnosed during pregnancy to allow time for delivery. One patient initially received Rituximab-methotrexate-cytarabine due to CNS infiltration while being treated with ABVD for Hodgkin lymphoma, pending review of the biopsy results. Thirty-four patients (62%) were treated with group B and 21 patients (38%) with group C protocols. Eight patients were escalated to group C due to an insufficient response on interim staging.

### 3.3. Outcome and Prognostic Factors

CR was achieved in 47 patients (85%), including most patients in group B (32, 94%) and 15 (71%) in group C. Among the eight patients who escalated from group B to group C, six (75%) achieved a complete response. The remaining two were refractory to subsequent therapy, eventually dying of disease progression.

**Figure 2:**
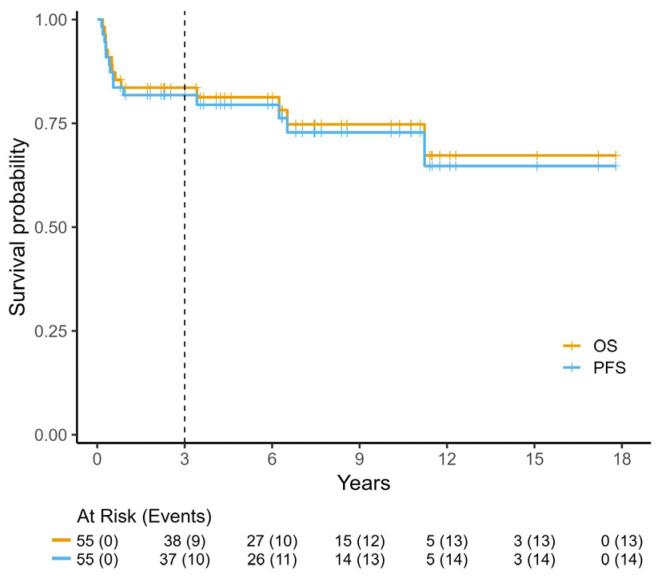
Progression-free (PFS) and overall survival (OS) of all patients.

**Figure 3:**
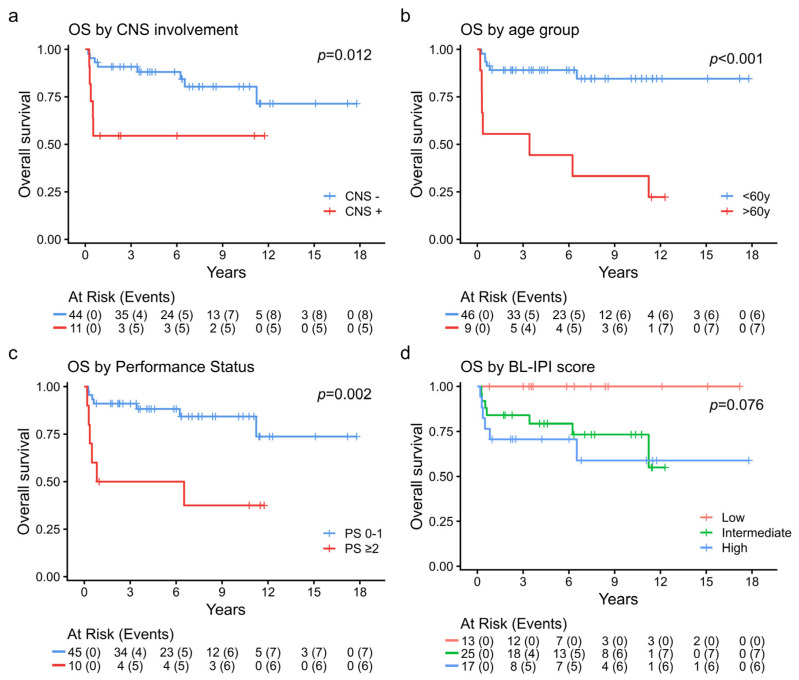
Overall survival (OS) by central nervous system (CNS) involvement (**a**), age (**b**), PS (**c**) and BL-IPI score (**d**).

With a median follow-up time of 7 years (range 0.2–17.8 years), PFS and OS at 3 years for all patients were 79% and 84%, respectively (Figure 2). Significant prognostic factors for OS included CNS involvement, age older than 60 and PS (Figure 3), whereas BL-IPI score had a trend for OS stratification. Thirteen patients (24%) died: 6 of progression, 3 of infectious complications (all older than 60 years old and in group C) and 4 of causes unrelated to lymphoma or chemotherapy at a median of 6.4 years after CR. Septic deaths occurred after RCOPADM2 in one patient and during CYVE maintenance in 2. All refractory patients died (five of disease progression and two of sepsis during salvage treatment). Another two patients relapsed within 6 months after obtaining a CR; one responded to salvage with HD-cytarabine and etoposide (CYVE) and maintenance cycles of group C, underwent allogeneic transplant, and is still alive, while the other did not respond to salvage and died of lymphoma within 3 months. Importantly, CNS involvement was absent in all patients at the time of relapse (*n* = 2) or persistent disease (*n* = 7). Two HIV+ patients responded to treatment and are still alive, and one died of refractory disease after 3 cycles.

### 3.4. Adverse Effects

**Figure 4:**
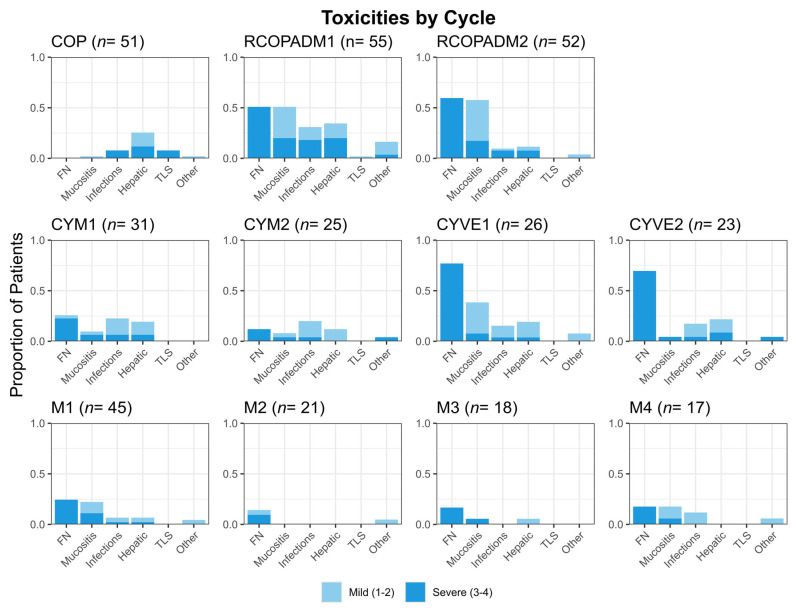
Toxicity description for each cycle. FN: febrile neutropenia; TLS: tumor lysis syndrome; other: cardiac, gastrointestinal or others.

Treatment toxicity per cycle is summarized in Figure 4. COP was associated with transient hepatic toxicity in 26% of cases and grade 3–4 tumor lysis syndrome (TLS) in 8%. During the first cycle of induction, only one patient experienced mild TLS. Overall, 282 episodes of febrile neutropenia (FN) and/or sepsis were recorded, including 16 cases (6%) of septic shock. The highest toxicity rates were observed during the induction (R-COPADM) and group C consolidation (CYVE) cycles, with mucositis occurring in up to 57% of patients during induction and FN reaching 77% during CYVE cycles. These treatment phases were also associated with non-neutropenic infections and elevated liver enzymes (Figure 4). In contrast, maintenance blocks were generally well tolerated; however, M1 was associated with infections in 30% of patients (including 24% cases of FN) and mucositis in 22%. Toxicity in older patients (>60 years) was particularly high, with grade 3-4 infections and mucositis in 53% and 20% of cycles, respectively.

Late sequelae were uncommon, including persistent grade 1–2 peripheral neuropathy (*n* = 2), recurrent infections (*n* = 2), atrial fibrillation with heart failure (*n* = 1), long-lasting cytopenias (up to the end of first year, *n* = 1) and grade 1 chronic renal failure (*n* = 1).

### 3.5. Feasibility and Health Resource Utilization

**Figure 5:**
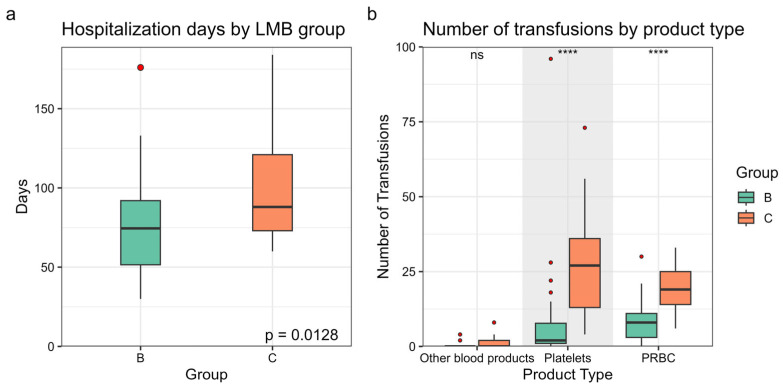
Health resource utilization. Transfusion requirements (**a**) and hospitalization time (**b**) stratified by LMB treatment group B and C. PRBCs: packed red blood cells. **** *p* < 0.0001. Red dots are outliers. Grade shadow is only to separate product types.

In most patients, the treatment duration exceeded the planned schedules of 95 days for group B and 196 days for group C, with a median overall delay of 15 days in group B and 22 days in group C (maximum delays of 55 and 88 days, respectively). Five patients finished earlier than planned (max. 16 days). The median hospitalization time was 74.5 days for group B and 88 days for group C (Figure 5a). Thirty patients (88%) in group B and 10 patients (48%) in group C completed the full planned protocol. Patients under 40 years of age were more likely to complete the planned treatment compared to those over 40 (84% vs. 57%, *p* = 0.05). Among the five responders in group C who interrupted treatment because of toxicity, two were de-escalated to less intensive regimens (R-CHOP and DA-EPOCH-R) after 3 and 4 cycles, while three discontinued treatment entirely after receiving 6, 7, and 7 cycles. Notably, none of these patients experienced relapse.

**Figure 6:**
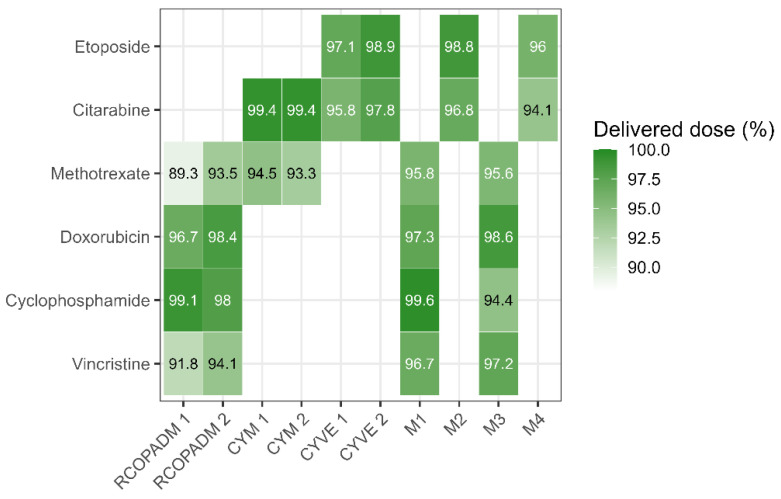
Cumulative delivered dose for each drug (excluding omitted cycles). Methotrexate was the most frequently adjusted drug.

A total of 60 treatment courses (16%) required dose reductions. The overall delivered dose intensity (for administered cycles) is illustrated in Figure 6. Patients over 60 years required greater dose reductions compared to younger patients (mean reduction 13.1% vs. 2.5%, *p* = 0.01) and more frequently experienced treatment interruptions (3 cases, 33%).

A total of 1604 transfusions were administered, including platelets, packed red blood cells, and other blood products. The median number of transfusions per patient was 19 (IQR 8.5–40.5; range 0–126). As expected, group C patients received significantly more blood products than group B (48.6 vs. 17.1, *p* ≤ 0.001) (Figure 5b).

### 3.6. CNS Infiltration

Eleven patients had CNS infiltration at diagnosis, all with isolated leptomeningeal involvement. Patients with CNS involvement more frequently had PS scores of 2 or more, B symptoms, and BM and liver infiltration (*p* < 0.02). As shown in Figure 3a, OS was significantly reduced in CNS positive patients (55% at 2 years), and deaths were mainly due to refractory disease (4 out of 5).

## 4. Discussion

To the best of our knowledge, this is the largest series of adult BL patients treated with LMB regimen outside clinical trials. At our institution we introduced some modifications in the original treatment regimen, including the addition of rituximab to the original LMB95 protocol and the omission of radiotherapy for patients with CNS infiltration during maintenance in order to avoid neurological toxicity.

Survival of adults with BL has significantly increased since the introduction of rituximab and the adoption of pediatric regimens that are of high intensity but short duration. With these protocols, such as HYPERCVAD, R-CODOX-M/IVAC, Burkimab, and LMB, survival has been reported to be between 72% and 90% at 3 years [5,6,7,8,19,20,21]. All these regimens include high doses of MTX and cytarabine, intrathecal prophylaxis, and multidrug combinations administered in rapid sequence to avoid the development of chemotherapy resistance. In our study we observed high CR rates (85%) with prolonged survival (84% at 3 years), in line with clinical trial results. The LMB protocol is relatively complex, particularly for patients stratified in group C, involving prolonged hospitalization. Real-world data on the use of this protocol is limited to a few small studies, reporting 5-year OS rates between 75% to 89%, and treatment-related mortality of 10%, as well as higher rates of infections and poorer outcomes in older patients [22,23,24].

Patients with CNS infiltration, which occurs in 10–30% of cases, consistently showed significantly poorer outcomes (OS of 49% at 3 years in a large series). [2,3] These unfavorable results have been observed across treatment regimens, particularly with DA-EPOCH-R, which lacks high-dose methotrexate (HD-MTX) [4,9]. Ferreri et al. recently published results from a 2-month intensive protocol (“CARMEN”), reporting 5-year PFS and OS rates of 72% and 76%, respectively [25]. In this study, among nine patients with CNS involvement at diagnosis (eight with Burkitt lymphoma), only five were alive at last follow-up (56%). Our results are in line with these findings, with an OS at 3 years of 55% for patients with CNS infiltration, with a plateau after the first year (Figure 3a). The omission of radiotherapy in the current series did not appear to negatively impact outcomes.

Overall, CNS relapses are rare with intensive treatments (<10% reported, none in our study); however, despite treatment intensification and CNS-directed strategies, patients with CNS involvement at diagnosis continue to experience poorer outcomes [3].

Toxicity with these intensive regimens is a major concern, in particular infectious complications, which lead to dose reductions, treatment interruptions and treatment related mortality [10,19,26]. Although adverse effects are frequently not well detailed in other studies, it would seem that LMB protocol group C is associated with higher toxicity rates than similar protocols. One likely reason is the length of the treatment, which includes 8 chemotherapy cycles. In our study, we examined toxicity by cycle and found that the induction phase and group C consolidation phase were the most toxic, whereas group B cytarabine-MTX blocks (CYM) and maintenance cycles were generally well tolerated. The fourth cycle of maintenance (M4) was still associated with a relatively high proportion of FN and infections (29%, Figure 4), probably due to the cumulative hematological toxicity. When compared with the LMB02 study, the overall rate of grade 3/4 mucositis was similar (9%). However, the incidence of infections was higher in our study (41% vs. 31%). We observed 3 toxic deaths (5.5%), whereas 26 patients (10%) died of non-lymphoma causes in the LMB02 study. These differences may be partly explained by the smaller number of patients in our cohort (55 vs. 257) and the older median age (47 vs. 34 years) in the clinical trial [7].

Overall, hematological and infectious toxicity were the main drivers of dose reductions (16%) and treatment interruptions (27%). Remarkably, delayed toxicity and sequela were uncommon. Namely, we did not observe second neoplasms (including myelodysplastic syndromes), although longer follow up is desirable.

In addition, the LMB protocol was associated with substantial health care resource utilization, evidenced by the high transfusion requirements (1604 transfusions for all patients) and the prolonged hospitalization times (4696 hospital days in total). Most HD-MTX containing regimens share a comparable supportive care requirements [10,27]. However, differences in protocol design and the application of risk-adapted treatment strategies (such as those used in the LMB regimen) make direct comparisons difficult.

Older BL patients consistently showed an inferior survival rate across studies, mainly due to toxicity [2,8,28,29,30]. Our study was not the exception, although the number of patients older than 60 years old was small. Even so, infections and severe mucositis in those patients were frequent; three of nine patients died of septic complications and another three died during later follow-up. Additionally, 3 of these patients required de-escalation to less intensive regimens.

An alternative to intensive regimens is the DA-EPOCH-R protocol, which has no systemic high-dose MTX or cytarabine. Two studies showed good efficacy of this protocol in different patient populations, with manageable toxicity, shorter hospitalization times and lower transfusion requirements [9,27]. On the other hand, this regimen was associated with higher CNS relapse rates and inferior outcomes in patients with CNS infiltration [4]. Taking these results into account, DA-EPOCH-R is an attractive option for low-risk patients according to the BL-IPI and for patients without CNS involvement.

In our study, five patients interrupted treatment because of toxicity, and none of them relapsed, raising the question of whether such long protocols (with four maintenance courses) are necessary. In this regard, Ribera et al. recently reported the results of the prospective BURKIMAB14 trial, which assessed the feasibility of chemotherapy dose reduction after two cycles in younger patients (≤55 years), achieving a complete metabolic response (CMR) [28]. Outcomes and toxicity were compared with the previous BURKIMAB08 protocol. Despite some limitations, the results were comparable in terms of efficacy, with a lower mortality rate among patients in CMR (5% vs. 11%), a reduced incidence of grade >2 infections, and a shorter duration of thrombocytopenia in the BURKIMAB14 cohort. Again, in patients older than 55 years, despite upfront dose reduction, treatment-related deaths were significantly higher (11/39 patients), particularly in early phases due to infections.

## 5. Conclusions

In summary, the LMB protocol demonstrated high survival rates in adults with Burkitt lymphoma but was associated with significant toxicity and considerable health resource utilization. Although treatment for patients stratified in group B was better tolerated, it still carried substantial risks of infection, prolonged hospitalization, and frequent transfusion requirements. Based on these findings, we do not recommend this regimen for older patients or those with low-risk disease (low BL-IPI). Importantly, outcomes remain poor in patients with CNS involvement despite treatment intensification, highlighting the urgent need for alternative therapeutic strategies in this subgroup.

## Figures and Tables

**Table 1:** Patients’ characteristics at diagnosis.

Characteristic	*n* = 55
Median age	34 (16–77)
Male Gender	38 (69%)
PS ≥ 2	10 (18%)
Stage III/IV	40 (73%)
Elevated LDH	41 (75%)
Albumin < 3 g/dL	6 (12%)
Tumor ≥ 10 cm	18 (33%)
HIV infection	3 (5.5%)
Extranodal disease (any site)	39 (71%)
Gastrointestinal	19 (35%)
Bone marrow	15 (28%)
CNS	11 (20%)
Liver	9 (16%)
BL-IPI group	
Low	13 (24%)
Intermediate	25 (45%)
High	17 (31%)
Treatment arm	
B	34 (62%)
C	21 (38%)

PS: performance status; CNS: central nervous system. BL-IPI: Burkitt Lymphoma International Prognostic Index.

## Supplementary Materials

The following supporting information can be downloaded at https://www.mdpi.com/article/10.3390/cancers17172914/s1, Table S1. Treatment courses utilized.; Table S2. Intrathecal chemotherapy scheme.
