# Supplementary material for: Intensive Treatment in Adult Burkitt Lymphoma with Lymphome Malin B (LMB) Regimen: Excellent Outcomes Despite Substantial Toxicity and Supportive Care Demands
Source: Cancers (Basel). 2025 Sep 5;17(17):2914. doi: 10.3390/cancers17172914 (PMC12427822; doi:10.3390/cancers17172914)
Supplement: Supplementary file 1 [file cancers-17-02914-s001.zip › cancers-3832146-supplementary.pdf]

Intensive Treatment in Adult Burkitt Lymphoma with Lymphome Malin B (LMB) Regimen: Excellent Outcomes Despite Substantial Toxicity and Supportive Care Demands

Supplementary material

Table S1. Treatment courses utilized.

	B	C (LMB95)	C (LMB02)	Days of administration
**COP**				
Cyclophosphamide	300 mg/m2			
Vincristine	1 mg/m2			
Prednisolone	60 mg/m2			
**R-COPADM1**				
Vincristine	1.4 mg/m2			D1
MTX	3 g/m2			D1
C1 / C2		3/8 g/m2		
C3 / C4		2/3 g/m2	3/3 g/m2	
C5 /C6		1/2 g/m2	2/3 g/m2	
Adriamycin	60 mg/m2			D2
Cyclophosphamide	0.5 g/m2			D2-D4
Prednisolone	60 mg/m2			
**R-COPADM2 (similar to 1, but)**				
Vincristine	1.4 mg/m2			D1&D6
Cyclophosphamide	1 g/m2			D2-D4
**CYM1-2**				
MTX	3 g/m2			D1
Cytarabine (cont. perf)	100 mg/m2			D2-D6
**CVYVE**				
HD-Cytarabine				D2-D5
C1 / C2		2/3 g/m2	3/3 g/m2	
C3 / C4		2/2 g/m2		
C5 /C6		1/1 g/m2	1.5/1.5 g/m2	
Etoposide		200 mg/m2		D2-D5

Cytarabine (cont. perf)		50 mg/m2		D1-D5
**M1-M3**				
Vincristine	2 mg			D1
HD-MTX*				D1
B	3 g/m2			
C1 / C2		3/8 g/m2		
C3 / C4		2/3 g/m2	3/3 g/m2	
C5 /C6		1/2 g/m2		
Adriamycin	60 mg/m2			D3
Cyclophosphamide	0.5 g/m2			D2&D3
Prednisolone	60 mg/m2			D1-D5
**M2-M4**				
Etoposide		150 mg/m2		D1-D3
Cytarabine		100 mg/m2		D1-D5

*HD-MTX and it-MTX in M3 were only in LMB02 protocol. Rituximab (375mg/m2) was administered with each cycle (with the exception of COP) in LMB95, while in LMB02 protocol was administered on day 0 and 6 of each R-COPADM. C1, C3 and C5 were used in patients with bone marrow infiltration without CNS involvement, while C2, C4 and C6 groups were used in CNS+ patients.

Table S2. Intrathecal chemotherapy scheme.

	**B**	**C1**	**C2**	**C3**	**C4**	**C5**	**C6**
**COP**	D1	D1, D5	D1, D3, D5	D1	D1, D3, D5	D1	D1, D3, D5
**R-COPADM 1 & 2**	D2, D6	D2, D6	D2, D4, D6	D2, D6	D2, D4, D6	D2, D6	D2, D6
**CYM 1 & 2**	D2, D6						
**CVYVE 1 & 2**		x	x	x	x	x	x
**M1-M3**	D2	D2	D2	D2	D2	x	x
**M2-M4**		x	x	x	x	x	x

The regimen consisted of methotrexate and hydrocortisone in group B, with cytarabine added to the combination in group C. D: day; x: no treatment.
